# Handbook of Natural Philosophy

**Published:** 1853-05

**Authors:** 


ART.—XV.—*Handbook of Natural Philosophy.* By DIONYSIUS LARDNER, D. C. L, Formerly Professor of Natural Philosophy and Astronomy in University College, London. Illustrated by upwards of 200 engravings on wood. Philadelphia: Blanchard and Lea, 1853. 12 mo., pp. 451.

The author of this little volume is extensively known as a lucid, forcible, well-informed writer on subjects of Natural Philosophy. In this work he treats on Heat, Magnetism and Electricity, common and voltaic, and in one which is to follow, purposes to treat of Astronomy and Meteorology. To such as have entered upon the active pursuits of business, and are still desirous "to sustain and improve their knowledge of the general truths of physics, and of those laws by which the stability and order of the material world are maintained," it must prove an acceptable manual. Of such persons there is a large number in our profession—men too busy with the active duties of life to read much general science, but too inqusitive to allow all its vast discoveries to pass by them unheeded; and for them a treatise like the one before us is just the work required. The table of contents is so full that no trouble will be experienced in turning to any subject discussed in it, at the same time that the language is so clear and the illustrations so perfect that every point will be easily understood.

